# Pharmacology of Antihistamines

**DOI:** 10.1097/1939-4551-4-S3-S22

**Published:** 2011-03-15

**Authors:** Diana S Church, Martin K Church

**Affiliations:** 1 University of Southampton School of Medicine, Southampton, UK; 2 Allergie-Centrum-Charité/ECARF, Charité-Universitätsmedizin, Berlin, Germany

**Keywords:** H_1_-antihistamines, cetirizine, levocetirizine, fexofenadine, loratadine, desloratadine

## Abstract

This article reviews the molecular biology of the interaction of histamine with its H_1_-receptor and describes the concept that H_1_-antihistamines are not receptor antagonists but are inverse agonists i.e. they produce the opposite effect on the receptor to histamine. It then discourages the use of first-generation H_1_-antihistamines in clinical practice today for two main reasons. First, they are less effective than second generation H_1_-antihistamines. Second, they have unwanted side effects, particularly central nervous system and anti-cholinergic effects, and have the potential for causing severe toxic reactions which are not shared by second-generation H_1_-antihistamines. There are many efficacious and safe second-generation H_1_-antihistamines on the market for the treatment of allergic disease. Of the three drugs highlighted in this review, levocetirizine and fexofenadine are the most efficacious in humans *in vivo*. However, levocetirizine may cause somnolence in susceptible individuals while fexofenadine has a relatively short duration of action requiring twice daily administration for full all round daily protection. While desloratadine is less efficacious, it has the advantages of rarely causing somnolence and having a long duration of action. Lastly, all H_1_-antihistamines have anti-inflammatory effects but it requires regular daily dosing rather than dosing 'on-demand' for this effect to be clinically demonstrable.

## 

It is now more than a century since the discovery of histamine,[1] more than 70 years since the pioneering studies of Anne Marie Staub and Daniel Bovet led to the discovery of the first antihistamine[2] and more than 60 years since the introduction into the clinic of antergan in 1942,[3] followed by diphenhydramine in 1945[4] and chlorpheniramine, brompheniramine, and promethazine later the same decade. Medicinal chemistry was very different in those days compared with the present day as elegantly described by Emanuel in his review entitled "Histamine and the antiallergic antihistamines: a history of their discoveries."[5] The usual way of testing novel compounds was to measure histamine-induced contractions of pieces of muscle from experimental animals, usually guinea-pig intestine, suspended in an organ bath. Candidate antihistaminic compounds were primarily modifications of those synthesized as cholinergic antagonists and are from diverse chemical entities, ethanolamines, ethylene diamines, alkylamines, piperazines, piperidines, and phenothiazines. It is hardly surprising, therefore, that these first-generation antihistamines had poor receptor selectivity and significant unwanted side effects.

During this time, knowledge of the nature and diversity of receptors was rudimentary to say the least and it was only several decades later that the existence of more than one species of histamine receptor was discovered. This review will concentrate on the histamine H_1_-receptor. Further details on the biology and clinical functions of histamine H_2_-, H_3_-, and H_4_-receptors are the subject of a separate review[6].

## The Histamine H_1_-Receptor

**Figure 1:**
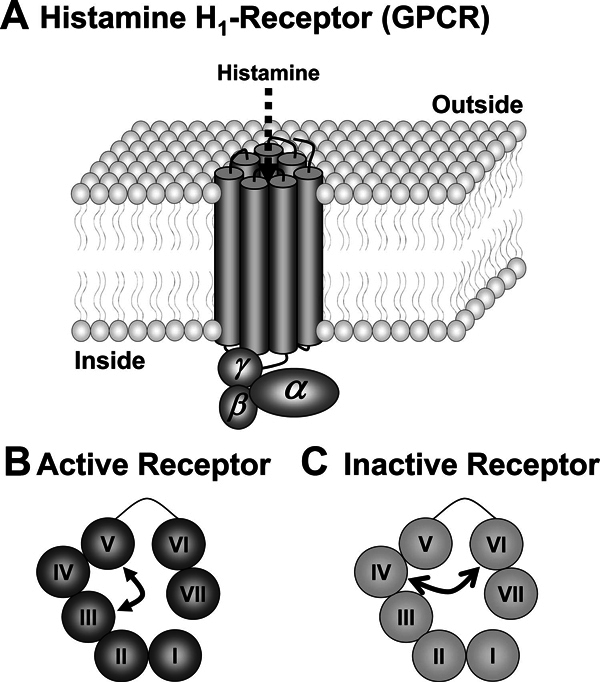
**A, Diagram of a histamine H1-receptor in a membrane showing the 7 transmembrane domains**. Histamine stimulates the receptor after its penetration into the central core of the receptor. B, A surface view of an activated receptor with histamine linking domains III and V. C, A surface view of an inactive receptor with cetirizine linking domains IV and VI.

The human histamine H_1_-receptor is a member of the superfamily of G-protein coupled receptors. This superfamily represents at least 500 individual membrane proteins that share a common structural motif of 7 transmembrane *α*-helical segments[7,8] (Figure 1A). The histamine H_1_-receptor gene encodes a 487 amino acid protein with a molecular mass of 55.8 kDa[9,10]. The absence of introns in the H_1_-receptor gene indicates that only a single receptor protein is transcribed with no splice variants[10].

The histamine H_1_-receptor, like other G-protein coupled receptors, may be viewed as "cellular switches," which exist as an equilibrium between the inactive or "off" state and the active or "on" state[11]. In the case of the histamine H_1_-receptor, histamine cross-links sites on transmembrane domains III and V to stabilize the receptor in its active conformation, thus causing the equilibrium to swing to the on position[12] (Figure 1B). H_1_-antihistamines, which are not structurally related to histamine, do not antagonize the binding of histamine but bind to different sites on the receptor to produce the opposite effect. For example, cetirizine cross-links sites on transmembrane domains IV and VI to stabilize the receptor in the inactive state and swing the equilibrium to the off position[13] (Figure 1C). Thus, H_1_-antihistamines are not receptor antagonists but are inverse agonists in that they produce the opposite effect on the receptor to histamine[14]. Consequently, the preferred term to define these drugs is "H_1_-antihistamines" rather than "histamine antagonists."

## First-Generation H_1_-Antihistamines

Because first-generation H_1_-antihistamines derive from the same chemical stem from which cholinergic muscarinic antagonists, tranquilizers, antipsychotics, and antihypertensive agents were also developed, they have poor receptor selectivity and often interact with receptors of other biologically active amines causing antimuscarinic, anti-*α*-adrenergic, and antiserotonin effects. But perhaps their greatest drawback is their ability to cross the blood-brain barrier and interfere with histaminergic transmission. Histamine is an important neuromediator in the human brain which contains approximately 64,000 histamine-producing neurones, located in the tuberomamillary nucleus. When activated, these neurones stimulate H_1_-receptors in all of the major parts of the cerebrum, cerebellum, posterior pituitary, and spinal cord[15] where they increase arousal in the circadian sleep/wake cycle, reinforce learning and memory, and have roles in fluid balance, suppression of feeding, control of body temperature, control of the cardiovascular system, and mediation of stress-triggered release of adrenocorticotrophic hormone and *β*-endorphin from the pituitary gland[16]. It is not surprising then that antihistamines crossing the blood-brain barrier interfere with all of these processes.

Physiologically, the release of histamine during the day causes arousal whereas its decreased production at night results in a passive reduction of the arousal response. When taken during the day, first-generation H_1_-antihistamines, even in the manufacturers' recommended doses, frequently cause daytime somnolence, sedation, drowsiness, fatigue, and impaired concentration and memory[17,18]. When taken at night, first-generation H_1_-antihistamines increase the latency to the onset of rapid eye movement sleep and reduce the duration of rapid eye movement sleep[19-21]. The residual effects of poor sleep, including impairment of attention, vigilance, working memory, and sensory-motor performance, are still present the next morning[20,22]. The detrimental central nervous system effects of first-generation H_1_-antihistamines on learning and examination performance in children and on impairment of the ability of adults to work, drive, and fly aircraft have been reviewed in detail in a recent review[23].

The use of first-generation H_1_-antihistamines in young children has recently been brought into question. In the United States, reports of serious and often life-threatening adverse events of promethazine in children led to a "boxed warning" being added in 2004 to the labeling of promethazine. The warning included a contraindication for use in children younger than 2 years and a strengthened warning with regard to use in children 2 years of age or older[24]. In February 2009, the Medicines and Healthcare products Regulatory Agency (MHRA) in the United Kingdom[25] advised that cough and cold remedies containing certain ingredients, including first-generation H_1_-antihistamines, should no longer be used in children younger than 6 years because the balance of benefit and risks has not been shown to be favorable. Reports submitted to regulators stated that more than 3000 people have reported adverse reactions to these drugs and that diphenhydramine and chlorpheniramine were mentioned in reports of 27 and 11 deaths, respectively[25].

## Second-Generation H_1_-Antihistamines

A major advance in antihistamine development occurred in the 1980s with the introduction of second-generation H_1_-antihistamines,[26] which are minimally sedating or nonsedating because of their limited penetration of the blood-brain barrier. In addition, these drugs are highly selective for the histamine H_1_-receptor and have no anticholinergic effects.

When choosing an H_1_-antihistamine, patients seek attributes that include good efficacy, a rapid onset of action, a long duration of action, and freedom from unwanted effects. Although some of these attributes may be predicted from preclinical and pharmacokinetic studies, it is only in the clinical environment that they may be definitively established.

### Efficacy

The efficacy of an H_1_-antihistamine is determined by 2 factors: the affinity of the drug for H_1_-receptors (absolute potency) and the concentration of the drug at the sites of the H_1_-receptors.

The affinity of an H_1_-antihistamine for H_1_-receptors is determined in preclinical studies. Desloratadine is the most potent antihistamine (Ki 0.4 nM) followed by levocetirizine (Ki 3 nM) and fexofenadine (Ki 10 nM) (the lower the concentration, the higher potency). Although these are often considered to be fixed values, they may be influenced by temperature and pH, and therefore, they can differ in physiologic and pathologic conditions. For example, in inflammation the pH of the tissues is reduced[27] from 7.4 to 5.8, leading to a 2-to 5-fold increase in the affinity of fexofenadine and levocetirizine for H_1_-receptors but no change in the affinity of desloratadine[28].

**Figure 2:**
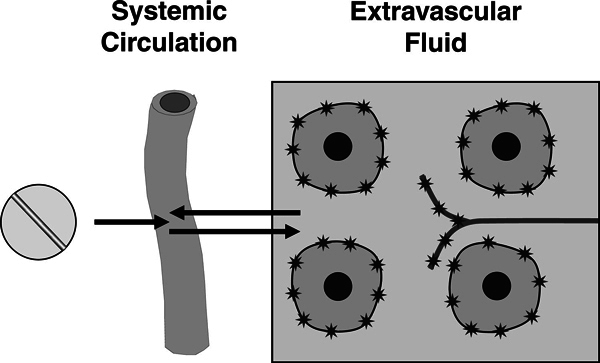
**Diagrammatic representation of the absorption of an H_1_-antihistamine**. Histamine H_1_-receptors are indicated by stars on the surface of cells and a sensory nerve in the extravascular space.

As shown in Figure 2, histamine receptors are situated on the cellular membranes of cells, including vascular and airways smooth muscle, mucous glands, and sensory nerves, all of which are surrounded by the extracellular fluid. Many factors affect concentration of free drug in this compartment. First, it must be absorbed into the systemic circulation after oral dosage with a tablet or capsule. Most H_1_-antihistamines are well absorbed, the exception being fexofenadine, which has a very variable absorption because of the influence of active transporting proteins as described later[29,30]. Second is the extent of plasma binding which, with H_1_-antihistamines, is high, varying from ~65% with desloratadine to ~90% for levocetirizine[31]. Third, and probably most influential, is the apparent volume of distribution which determines the plasma concentration of a drug after complete body distribution. The apparent volume of distribution is limited for levocetirizine (0.4 L/kg), larger for fexofenadine (5.4-5.8 L/kg), and particularly large for desloratadine (~49 L/kg)[32]. The large apparent volume of distribution of desloratadine is largely due to its extensive intracellular uptake. In the study of Gillard and colleagues,[31] the 4-hour plasma concentrations of levocetirizine, desloratadine, and fexofenadine are 28, 1, and 174 nM, respectively.

Because data on the concentrations of H_1_-antihistamines in relevant extracellular fluids is generally lacking, the best indirect estimate of efficacy is obtained by calculating receptor occupancy from knowledge of absolute potency and peak drug concentrations in the plasma, usually at ~4 hours after a single oral dose using the following equation[31].

$$\text{Receptor}\;\text{occupancy}\;\left(\%\right)={\text{B}}_{\text{max}}\times \frac{\text{L}}{\text{L}+\text{Ki}}$$

where *B*_max _is the maximal number of binding sites (set to 100%), *L *the concentration of free drug in the plasma, and Ki the equilibrium inhibition constant (≡ absolute potency).

Thus, the calculation of receptor occupancy after single oral doses of drug shows values of 95%, 90%, and 71% for fexofenadine, levocetirizine, and desloratadine, respectively, indicating that they are all very effective H_1_-antihistamines. Although receptor occupancy for these drugs appears to correlate with pharmacodynamic activity in skin wheal and flare studies and with efficacy in allergen challenge chamber studies,[33,34] are the differences relevant in clinical practice? Studies in allergic rhinitis suggest that the above 3 drugs are of similar effectiveness[35,36]. However, in chronic urticaria in which local histamine concentrations are high, the differences do seem to be important. For example, in head to head studies in this condition levocetirizine appears significantly more effective than desloratadine[37,38].

### Speed of Onset of Action

**Figure 3:**
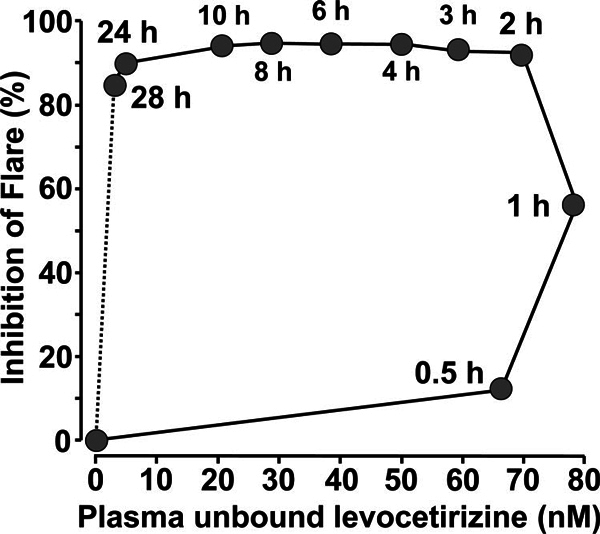
**Hysteresis loop of the inhibition of the histamine-induced flare response plotted against the plasma concentration of unbound levocetirizine after administration of a single 5-mg dose to children**. Redrawn from Ref. 39.

The speed of onset of action of a drug is often equated to the rate of its oral absorption. However, this is not strictly correct as seen from Figure 3, which shows the inhibition of the histamine-induced flare response (indicative of the prevention by levocetirizine of sensory neurone stimulation in the extravascular space) plotted against the concentration of free drug in the plasma. In this study in children,[39] plasma concentrations of drug are near maximum by 30 minutes and yet it takes an additional 1.5 hours for the drug to diffuse into the extravascular space to produce a maximal clinical effect. In adults, the maximal inhibition of the flare response is usually ~4 hours for levocetirizine, fexofenadine, and desloratadine[40-42] but may be longer for drugs, such as loratadine and ebastine, which require metabolism to produce their active moiety[40].

### Duration of Action

Figure 3 also shows that the duration of action of levocetirizine in inhibiting the histamine-induced flare response is also much longer than would be predicted from a knowledge of its plasma concentration[39]. This is presumably due to "trapping" of the drug by its strong and long-lasting binding to histamine H_1_-receptors[13]. Although less active in the wheal and flare test, desloratadine has a similarly long duration of action[41]. However, the duration of action of fexofenadine, calculated in the study of Purohit et al[43] as the time for the wheal to be inhibited by at least 70%, is less prolonged, being 8.5 hours for 120 mg fexofenadine compared with 19 hours for cetirizine. The primary reason for the shorter duration of action of fexofenadine is that it is actively secreted into the intestine and urine[44].

### Anti-Inflammatory Effects

Although the majority of research into H_1_-antihistamines has focused on the histamine-dependent early phase symptoms of the allergic response, it is now becoming clear that these drugs have anti-inflammatory effects. This follows the observation by Bakker and colleagues[45] that histamine can activate NF-κB, a transcription factor involved in the synthesis of many pro-inflammatory cytokines and adhesion molecules involved in the initiation and maintenance of allergic inflammation. The anti-inflammatory effects of H_1_-antihistamines, which is a class effect mediated through the H_1_-receptor, are summarized in Ref[14]. The clinical implications of this lie in the ability of H_1_-antihistamines to reduce nasal congestion and hyper-reactivity,[36] which result from the sensitization of sensory neurones in the nose by allergic inflammation[46]. However, as nasal congestion is more slowly relieved than other nasal symptoms,[47] continuous rather than on demand therapy with antihistamines is required for its treatment[48].

### Elimination

The metabolism and elimination of H_1_-antihistamines have been extensively reviewed elsewhere[32,49] and will be only briefly summarized here. Cetirizine and levocetirizine are not metabolized and are excreted primarily unchanged in the urine[32]. Desloratadine undergoes extensive metabolism in the liver. Although this gives the potential for drug-drug interactions, no significant interactions have been reported[49]. Fexofenadine, which is also minimally metabolized, is excreted primarily in the feces after its active secretion into the intestine under the influence of active drug-transporting molecules[49]. This gives the potential for interactions with agents such as grapefruit juice and St Johns Wort, which inhibit these transporters. Although plasma concentrations of fexofenadine may be increased by these agents, no significant resulting adverse reactions have been reported[49].

### Unwanted Effects

#### Somnolence

A major reason for the reduced penetration of second-generation H_1_-antihistamines into the brain is because their translocation across the blood-brain barrier is under the control of active transporter proteins, of which the ATP-dependent efflux pump, P-glycoprotein, is the best known[50,51]. It also became apparent that antihistamines differ in their substrate specificity for P-glycoprotein, fexofenadine being a particularly good substrate[52]. In the brain, the H_1_-receptor occupancy of fexofenadine assessed using positron emission tomography scanning is negligible, <0.1%, and, in psychomotor tests, fexofenadine is not significantly different from placebo[53]. Furthermore, fexofenadine has been shown to be devoid of central nervous effects even at supraclinical doses, up to 360 mg[54].

Although fexofenadine is devoid of CNS effects, other second-generation H_1_-antihistamines many still penetrate the brain to a small extent where they have the potential to cause some degree of drowsiness or somnolence, particularly when used in higher doses. For example, positron emission tomography scanning of the human brain has shown that single oral doses of 10 and 20 mg of cetirizine caused 12.5 and 25.2% occupancy of the H_1_-receptors in prefrontal and cingulate cortices, respectively[55]. These results would explain the repeated clinical findings that the incidence of drowsiness or fatigue is greater with cetirizine than with placebo[56-59]. Recent publications have suggested that, at manufacturer's recommended doses, levocetirizine is less sedating than cetirizine[60] and desloratadine causes negligible somnolence[49,61]. However, it should be pointed out that "mean results" do not reveal everything as some patients may show considerable somnolence whereas others are unaffected.

#### Cardiotoxicity

The propensity of astemizole and terfenadine to block the I_Kr _current, to prolong the QT interval, and to potentially cause serious polymorphic ventricular arrhythmias such as torsades de pointes is well documented[14,62]. These 2 drugs are no longer approved by regulatory agencies in most countries. In addition, some first-generation H_1_-antihistamines, such as promethazine,[63] brompheniramine,[64] and diphenhydramine,[65] may also be associated with a prolonged QTc and cardiac arrhythmias when taken in large doses or overdoses. No clinically significant cardiac effects have been reported for the second-generation H_1_-antihistamines loratadine, fexofenadine, mizolastine, ebastine, azelastine, cetirizine, desloratadine, and levocetirizine[66-69].

## Conclusions

In conclusion, the use of first-generation H_1_-antihistamines should be discouraged in clinical practice today for 2 main reasons. First, they are less effective than second-generation H_1_-antihistamines[17,70,71]. Second, they have unwanted side effects and the potential for causing severe toxic reactions which are not shared by second-generation H_1_-antihistamines. With regard to second-generation H_1_-antihistamines, there are many efficacious and safe drugs on the market for the treatment of allergic disease. Of the 3 drugs highlighted in this review, levocetirizine and fexofenadine are the most potent in humans in vivo. However, levocetirizine may cause somnolence in susceptible individuals whereas fexofenadine has a relatively short duration of action and may be required to be given twice daily for all-round daily protection. Although desloratadine is less potent, it has the advantages of rarely causing somnolence and having a long duration of action. Lastly, all H_1_-antihistamines have anti-inflammatory effects but it requires regular daily dosing rather than dosing "on demand" for this action to be clinically demonstrable.
